# Caveolin-1 expression and stress-induced premature senescence in human intervertebral disc degeneration

**DOI:** 10.1186/ar2468

**Published:** 2008-08-05

**Authors:** Sarah Kathleen Heathfield, Christine Lyn Le Maitre, Judith Alison Hoyland

**Affiliations:** 1 Tissue Injury and Repair Group, Research School of Clinical and Laboratory Sciences, Faculty of Medical and Human Sciences, Stopford Building, The University of Manchester, Oxford Road, Manchester, M13 9PT, UK; 2 Biomedical Research Centre, Biosciences, Faculty of Health and Wellbeing, Sheffield Hallam University, City Campus, Howard Street, Sheffield, S1 1WB, UK

## Abstract

**Introduction:**

Chronic and debilitating low back pain is a common condition and a huge economic burden. Many cases are attributed to age-related degeneration of the intervertebral disc (IVD); however, age-related degeneration appears to occur at an accelerated rate in some individuals. We have previously demonstrated biomarkers of cellular senescence within the human IVD and suggested a role for senescence in IVD degeneration. Senescence occurs with ageing but can also occur prematurely in response to stress. We hypothesised that stress-induced premature senescence (SIPS) occurs within the IVD and here we have investigated the expression and production of caveolin-1, a protein that has been shown previously to be upregulated in SIPS.

**Methods:**

Caveolin-1 gene expression in human nucleus pulposus (NP) cells was assessed by conventional and quantitative real-time polymerase chain reaction (PCR), and caveolin-1 protein expression was examined within human IVDs using immunohistochemistry. The correlation between caveolin-1 and p16^INK4a ^(biomarker of cellular senescence) gene expression was investigated using quantitative real-time PCR.

**Results:**

Caveolin-1 gene expression and protein expression were demonstrated within the human IVD for the first time. NP cells from degenerate discs exhibited elevated levels of caveolin-1 which did not relate to increasing chronological age. A negative correlation was observed between gene expression for caveolin-1 and donor age, and no correlation was found between caveolin-1 protein expression and age. A positive correlation was identified between gene expression of caveolin-1 and p16^INK4a^.

**Conclusion:**

Our findings are consistent with a role for caveolin-1 in degenerative rather than age-induced changes in the NP. Its expression in IVD tissue and its association with the senescent phenotype suggest that caveolin-1 and SIPS may play a prominent role in the pathogenesis of IVD degeneration.

## Introduction

Low back pain (LBP) is a condition that affects a significant proportion of the population, with a lifetime incidence rate in excess of 70% in industrialised nations [1]. It not only impacts on quality of life, but also places a substantial financial burden on the National Health Service and the economy in general due to loss of working days [1,2]. Many cases of LBP are attributed to degeneration of the intervertebral disc (IVD) and imaging studies have indicated a link between IVD degeneration and LBP [3,4].

To date, no clear mechanism for IVD degeneration has been identified, although the involvement of both environmental and genetic factors has been proposed [5-8]. The occurrence of IVD degeneration increases with age [9,10]; however, a subset of individuals appear to exhibit accelerated degeneration that is independent of age [5,6]. This has led to speculation that additional factors could play a key role in the development of degeneration in some individuals.

There is increasing evidence that many features of IVD degeneration, including altered matrix synthesis and enhanced matrix degradation, originate at a cellular level [6,11,12]. Cellular senescence is a strong candidate for the prolonged alteration in cellular activity observed during degeneration. Senescence and accompanying alterations in cell function have been implicated in ageing-related, degenerative, and pathological changes in a variety of tissues, including atherosclerotic plaque development within blood vessels and osteoarthritic alterations to cartilage [13-15]. Two groups have shown increased staining for senescence-associated β-galactosidase (SA-β-gal) in cells from prolapsed and degenerate IVD cells, respectively, when compared with non-degenerate discs [16,17]. More recently, our group has presented more comprehensive evidence of senescence biomarkers in human IVD samples, demonstrating increased cellular senescence during IVD degeneration [18]. In particular, cells from degenerate discs exhibited increased SA-β-gal activity, elevated expression of the cell cycle inhibitor p16^INK4a^, telomere erosion, and a decrease in replicative potential. Furthermore, a correlation was observed between p16^INK4a ^expression and the expression of matrix-degrading enzymes matrix metalloproteinase (MMP)-13 and a disintegrin and metalloproteinase with thrombospondin motifs (ADAMTS)-5, suggesting a role for cell senescence in the molecular processes observed during IVD degeneration [18].

Senescence occurs naturally with ageing but can also occur prematurely in response to stresses (such as exposure to cytokines or oxidative stress) in a number of cell types [19-24]. Since telomeric erosion and p16^INK4a ^protein expression are increased in degenerate discs compared to non-degenerate age-matched samples [18], we hypothesised that stress-induced premature senescence (SIPS) occurs within the IVD and may be responsible for the accelerated degeneration observed in some individuals.

Caveolae are plasma membrane compartments found abundantly in terminally differentiated cells such as fibroblasts and endothelial and muscle cells [25]. The mammalian caveolin gene family codes for three 21 to 25 kDa caveolin proteins, which are integral membrane proteins essential for the structural integrity and function of caveolae [26]. Expression of caveolin-3 is muscle-specific, whereas caveolin-1 and caveolin-2 are coexpressed in many cell types [26]. Proposed functions include lipid transport, membrane trafficking, and a role in intracellular signalling pathways which stems from the colocalisation of caveolins with a variety of signal transduction molecules [25-28]. Interestingly, caveolin-1 has been implicated in the senescent phenotype of several cell types, including human fibroblasts, lung adenocarcinoma cells, endothelial cells, and articular chondrocytes [19,29-33]. Moreover, caveolin-1 has been proposed to mediate SIPS in murine fibroblasts and human articular chondrocytes in response to oxidative stress and the inflammatory cytokine interleukin-1β (IL-1β) (both of which are known to be increased during IVD degeneration) [19,31,34-38]. Here, we have investigated the expression of caveolin-1 in human IVDs and correlated its expression with the cell cycle inhibitor and the biomarker of senescence p16^INK4a^, focusing on the nucleus pulposus (NP) as this area shows the most evidence of cell senescence in human IVDs [18].

## Materials and methods

### Tissue samples

Human IVD tissue was obtained either at post mortem (PM) examination or from patients undergoing surgery, where patients were selected on the basis of magnetic resonance imaging-diagnosed degeneration and progression to anterior resection either for spinal fusion or disc replacement surgery for chronic LBP. Local research ethics committee approval was obtained together with informed consent from the patient or relatives. Disc tissue was removed as detailed previously [37].

#### General procedure for tissue specimens

A block of tissue (incorporating annulus fibrosus [AF] and NP in continuity) was fixed in 10% vol/vol neutral buffered formalin and embedded in paraffin wax. Four micron sections were stained with haematoxylin and eosin to grade the degree of morphological degeneration according to previously published criteria that assess the demarcation between NP and AF, proteoglycan content of the NP, presence and extent of structural fissures, and cell cluster formation [39]. Potential grades range between 0 and 12. A grade of 0 to 3 indicates a histologically non-degenerate IVD, 4 to 7 indicates evidence of intermediate (or moderate) degeneration, and 8 to 12 indicates severe degeneration. Further tissue sections were taken for immunohistochemical analysis of caveolin-1.

### Isolation of nucleus pulposus cells

To obtain NP cells from human IVD tissue, NP tissue was identified and dissected from AF. NP tissue was finely chopped and digested in a solution of 2 U/mL protease (Sigma-Aldrich, Gillingham, UK) in Dulbecco's modified Eagle's medium plus Ham's F-12 nutrient medium (DMEM + F-12) (Gibco BRL, now part of Invitrogen, Paisley, UK) for 30 minutes at 37°C. NP cells were washed twice with DMEM + F-12 prior to cell isolation with collagenase type I treatment (0.4 mg/mL; Invitrogen).

### Conventional reverse transcription-polymerase chain reaction

To investigate gene expression of caveolin-1 in human NP cells, RNA was extracted from isolated cells following the standard procedure for TRIzol^® ^reagent (Invitrogen). cDNA was then synthesised using Superscript II in accordance with the instructions of the manufacturer (Invitrogen). A standard Platinum Taq (Invitrogen) method was used for conventional polymerase chain reaction (PCR), using a concentration of 1.5 mM MgCl_2_. Primers specific for caveolin-1 [19] and the housekeeping gene *18S *(Invitrogen) are detailed in Table 1. All primers were confirmed for gene specificity using BLAST (Basic Local Alignment Search Tool) (Genbank database sequences). Reactions, including non-template controls, were conducted for 35 cycles, including the annealing temperature of 58°C on a thermal cycler (MJ Research, now part of Bio-Rad Laboratories, Hercules, CA, USA), and products were analysed alongside a 100-base pair DNA ladder (Hyperladder IV; Bioline, London, UK) by electrophoresis on a 1.5% wt/vol agarose gel containing 0.2 μg/mL ethidium bromide (Sigma-Aldrich). Product bands were visualised by UV transillumination and images were captured using Gene Snap software (Syngene, Cambridge, UK).

**Table 1:** Details of polymerase chain reaction (PCR) primers, probes, and amplicon sizes

Conventional PCR conditions			
Target	Forward primer 5' to 3'	Reverse primer 5' to 3'	Amplicon size, base pairs (bp)
*18S*	GCC ATG CAT GTC TAA GTA CG	GCT GGC ACC AGA CTT GCC	574 bp
Caveolin-1	AAG GAG ATC GAC CTG G	GGA ATA GAC ACG GCT G	309 bp

Real-time PCR primers and probes			

Target	Forward primer 5' to 3'	Probe 5' to 3'	Reverse primer 5' to 3'
*18S*	PDAR	PDAR (VIC-TAMRA)	PDAR
Caveolin-1	ACT TGC AAC CGT CTG TTA TGC T	FAM – ACA TGG CCC CTC CCC – MGB	GCA AAG GGA TGC TTG GAT TAG GT
p16^INK4a^	GGC TCT ACA CAA GCT TCC TTT CC	FAM – ACC CTG GCT CTG ACC A – MGB	TCA TGA CCT GCC AGA GAG AAC A

PDAR, pre-developed assay reagent.

### Quantitative real-time polymerase chain reaction

Quantitative real-time reverse transcription-PCR (qRT-PCR) was performed to further examine caveolin-1 gene expression in human NP cells and to investigate any correlation between caveolin-1 and p16^INK4a ^gene expression in isolated NP cells using the standard curve method of analysis as described previously [18].

#### Primers and probe design

Primers and FAM-MGB probe specific for human caveolin-1 were designed by Applied Biosystems (ABI) (Warrington, UK) upon provision of caveolin-1-specific exon sequence (Gene expression assays) (Table 1). p16^INK4a ^primers and probe were as described previously [18], and *18S *primer/VIC-TAMRA probe set was a pre-developed assay reagent (PDAR) purchased from ABI.

#### Genomic curve standards

Genomic DNA (gDNA) was used to create standard curves for absolute quantification of copy number per reaction. gDNA (Promega Corporation, Southampton, UK) was homogenised, diluted to 100 ng/μL, and sonicated on ice. Serial dilutions of gDNA were prepared to generate standards with gene copy numbers of 75,000, 7,500, 750, 75, and 0 copies per 25 μL reaction.

#### Quantitative real-time reverse transcription-polymerase chain reaction amplification

qRT-PCRs were carried out in triplicate in a 96-well plate. Reactions contained 12.5 μL of mastermix (Taqman^® ^Universal PCR mastermix; ABI) and 2.5 μL of template cDNA or gDNA. Primers were added to a final concentration of 900 nM and probe to a concentration of 250 nM, and molecular-grade water was added to a total reaction volume of 25 μL. A gDNA standard curve for each gene was included on each plate. Real-time PCR was performed using an ABI Prism 7000 sequence detection system (ABI). Reactions consisted of an initial Taq activation step of 95°C for 10 minutes to denature DNA and activate Taq polymerase followed by 40 cycles of 95°C for 15 seconds and 60°C for 1 minute.

#### Quantitative real-time reverse transcription-polymerase chain reaction analysis

Following amplification, an auto-baseline was set using the ABI 7000 sequence detection software and a threshold was set for each gene, above background levels and within the exponential phase. From these, a cycle threshold (Ct) was obtained for each well and data exported into Microsoft Excel (Microsoft Corporation, Redmond, WA, USA), where the three Ct values for each sample were averaged. Data were analysed as described previously [18] and results were expressed as copy number of target gene per 100 ng cDNA normalised to *18S*.

### Immunohistochemistry

Immunohistochemistry (IHC) was used to determine the expression and localisation of caveolin-1 protein in the NP of 28 paraffin-embedded disc samples (Table 2). Normal human skin tissue was used as a positive control. The protocol was based upon previously published IHC [40]. Briefly, following deparaffination, blocking of endogenous peroxidase activity, and enzyme retrieval in 0.01% wt/vol chymotrypsin (Sigma-Aldrich) solution at 37°C for 20 minutes, sections were washed and incubated with 25% rabbit serum (Sigma-Aldrich) to block non-specific binding sites. Sections were then incubated at 4°C overnight with mouse monoclonal antibody against human caveolin-1 (BD Transduction Laboratories catalogue number 610406, clone 2297; BD Biosciences, Oxford, UK) (1:10 dilution in 25% rabbit serum in 0.1% bovine serum albumin; Sigma-Aldrich). Negative control sections were incubated with an equivalent concentration of mouse IgG1 (Dako UK Ltd., Ely, UK). Following washes in Tris-buffered saline (TBS), sections were incubated with biotinylated rabbit anti-mouse antiserum (1:400; Dako UK Ltd.) for 30 minutes at room temperature. After further washes in TBS, immunoreactivity was visualised using the streptavidin-biotin complex (Dako UK Ltd.) technique with 3,3'-diaminobenzidine tetrahydrochloride solution (Sigma-Aldrich). Sections were subsequently rinsed in water, counterstained with Mayer's haematoxylin, dehydrated, and mounted with Pertex (HistoLab, Gothenburg, Sweden).

**Table 2:** Details of human nucleus pulposus samples used to study caveolin-1 protein expression by immunohistochemistry

Laboratory number	Histological grade	Age, years	Source
1	1	25	Surgery
2	1	30	PM
3	1	47	PM
4	2	47	PM
5	2	75	PM
6	2	Unknown	PM
7	3	30	PM
8	3	30	PM
9	3	37	PM
10	3	74	PM
11	4	30	PM
12	4	37	PM
13	5	30	PM
14	5	74	PM
15	5	Unknown	PM
16	5	Unknown	PM
17	6	74	PM
18	6	75	PM
19	7	75	PM
20	7	78	PM
21	8	58	PM
22	8	75	PM
23	9	58	PM
24	9	74	PM
25	9	74	PM
26	10	58	PM
27	11	46	Surgery
28	12	Unknown	PM

PM, post mortem tissue.

Sections were visualised using a Leica RMDB microscope (Leica Camera Limited, Knowlhill, Milton Keynes, UK), and images were captured using a digital camera and Bioquant Nova image analysis system (Bioquant Image Analysis Corporation, Nashville, TN, USA). For analysis, the NP was identified morphologically within each disc section. Within each section, a minimum of 200 NP cells were analysed from at least five different fields of view and immunopositivity was calculated as a percentage of the total cell population.

### Statistical analysis

Data were non-parametric and thus Mann-Whitney *U *tests were conducted to compare gene copy number and numbers of caveolin-1-immunopositive cells in non-degenerate NP (grades 0 to 3) and degenerate NP (grades 4 to 7 and 8 to 12). Non-parametric linear regression analysis was performed to analyse the correlation between copy numbers of different genes and between gene copy numbers and subject age or number of caveolin-1-immunopositive cells and subject age.

## Results

### Caveolin-1 gene expression in human nucleus pulposus cells

**Figure 1:**
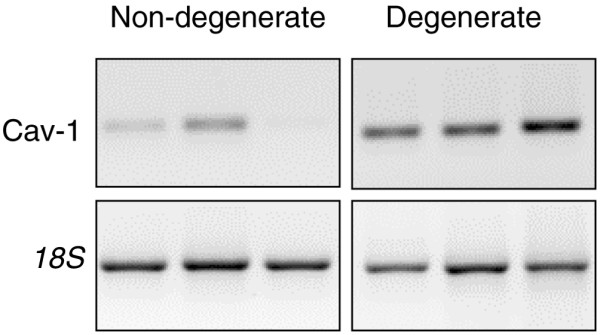
Conventional reverse transcription-polymerase chain reaction (RT-PCR) for caveolin-1 and housekeeping gene *18S*. Representative photographs following agarose gel electrophoresis of products from conventional RT-PCR for caveolin-1 and *18S*. cDNA samples displayed are non-degenerate samples from a post mortem (PM) source (respective grades [G] and ages of subjects: G3, 30 years; G1, 30 years; and G2, 75 years) and degenerate samples from surgical and PM sources (G5, 29 years; G6, 34 years; and G9, 74 years). Photographs are inverted to improve visualisation of product bands. Cav-1, caveolin-1.

**Figure 2:**
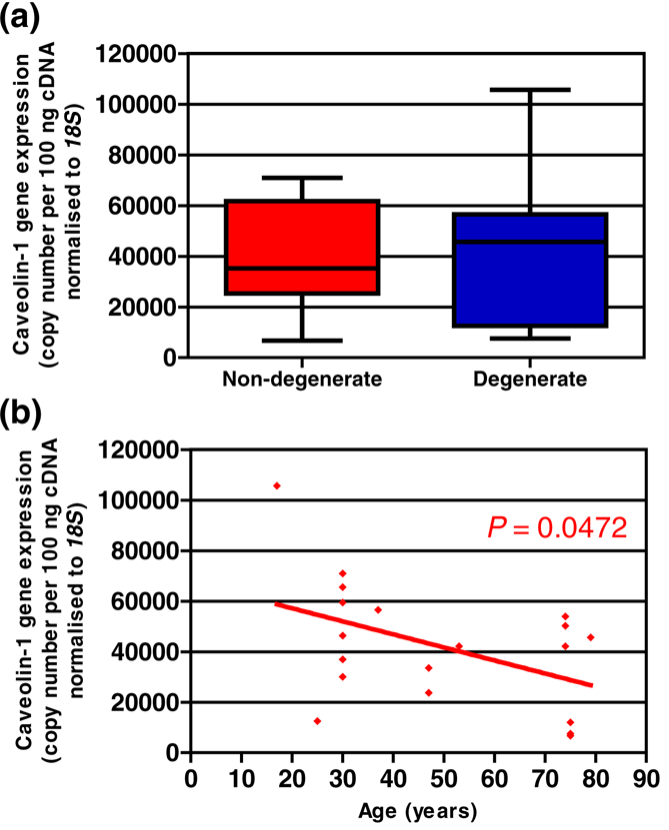
Quantitative real-time reverse transcription-polymerase chain reaction analysis of caveolin-1 gene expression levels in nucleus pulposus (NP) cells from human intervertebral disc. **(a) **Caveolin-1 gene expression per 100 ng cDNA normalised to *18S *in non-degenerate and degenerate NP presented as box-and-whisker plot (5–95 percentile). **(b) **Correlation of caveolin-1 gene expression with age of subject. Non-parametric linear regression analysis (*P *= 0.0472; *R*^2 ^= 0.2122).

cDNAs derived from cells directly extracted from the NP of 19 different IVDs, from both PM and surgical sources, were analysed for expression of the caveolin-1 gene. Eight samples were taken from non-degenerate IVD (grades 0 to 3; mean age ± standard deviation [SD] 45.4 ± 18.7 years) and 11 samples from degenerate IVD (grades 4 to 9; 51.7 ± 24.3 years). Gene expression for caveolin-1 was detected in the NP tissue of every sample analysed (qRT-PCR analysis). Comparison of caveolin-1 gene expression by non-degenerate and degenerate samples demonstrated higher gene expression in degenerate samples (conventional RT-PCR analysis, Figure 1). This was supported by qRT-PCR analysis (Figure 2a) in that non-degenerate samples demonstrated a median caveolin-1 gene copy number of 35,220 with a range of 6,740 to 70,9222 copies per 100 ng cDNA compared with the elevated degenerate median caveolin-1 gene copy number of 45,695 with a range of 7,589 to 105,626 copies per 100 ng cDNA (Figure 2a). A negative correlation was observed between gene expression for caveolin-1 and age of the donor (*P *= 0.0472) (Figure 2b).

### Immunohistochemical detection of caveolin-1 protein in human nucleus pulposus

**Figure 3:**
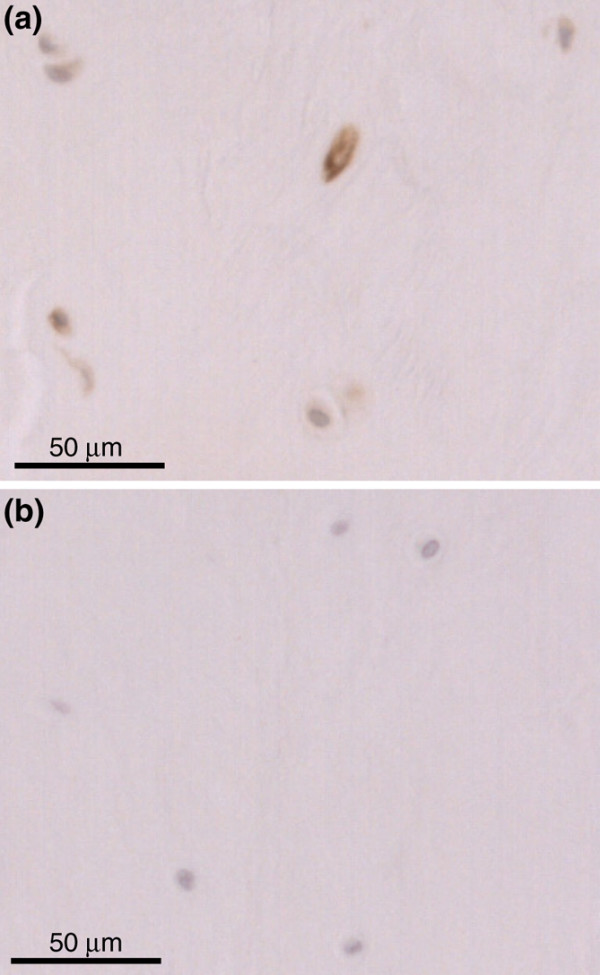
Caveolin-1 immunohistochemistry. **(a) **Photomicrograph demonstrating staining for caveolin-1 protein in degenerate human nucleus pulposus (sample 28). **(b) **Immunoglobulin G controls were negative.

**Figure 4:**
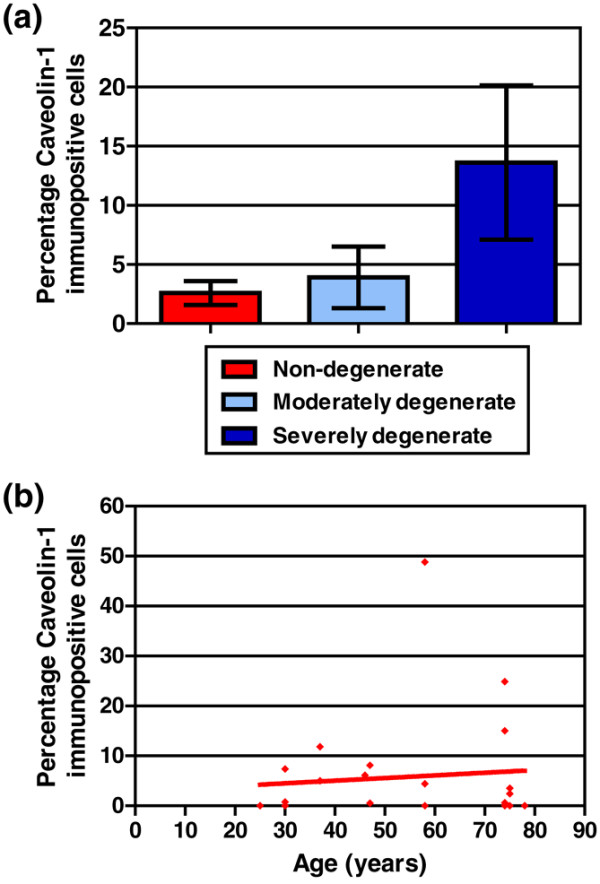
Analysis of caveolin-1 immunohistochemistry. **(a) **Percentage of cells immunopositive for caveolin-1 protein in non-degenerate, moderately degenerate, and severely degenerate intervertebral discs. Data are shown as mean ± SEM. **(b) **Correlation of caveolin-1 protein expression with age of subject. Non-parametric linear regression analysis (*P *= 0.6609; *R*^2 ^= 0.0089).

Caveolin-1 protein expression was investigated in 28 IVD samples (for sample details, see Table 2). Immunohistochemical analysis for caveolin-1 demonstrated cytoplasmic/membrane staining within the chondrocyte-like cells of the NP (Figure 3). The percentage of immunopositive cells for caveolin-1 increased from 2.59% ± 1.01% (mean ± standard error of the mean [SEM]) in non-degenerate discs to 13.62% ± 6.51% in severely degenerate samples (Figure 4a). All IgG1 controls were negative. It must be noted that the majority of patients with severely degenerate discs were above 50 years of age; however, in the 24 samples of all grades for which the chronological age of individuals was known, no correlation was observed between caveolin-1 immunopositivity and age of the donors (*P *= 0.6609) (Figure 4b).

### Correlation between caveolin-1 gene expression and gene expression of the senescence biomarker p16^INK4a^

**Figure 5:**
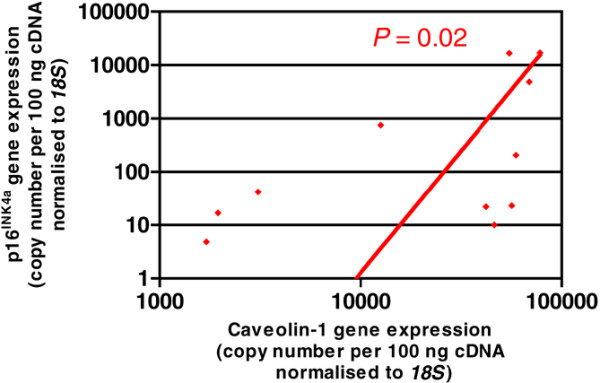
Correlation between caveolin-1 and p16^INK4a ^gene expression in degenerate nucleus pulposus samples. Caveolin-1 and p16^INK4a ^gene expression (copy number per 100 ng cDNA normalised to *18S*) analysed by quantitative real-time reverse transcription-polymerase chain reaction. Non-parametric linear regression analysis (*P *= 0.02; *R*^2 ^= 0.4725).

Seventeen NP samples were analysed for both caveolin-1 and p16^INK4a ^gene expression using qRT-PCR. Analysis of p16^INK4a ^expression agreed with our previous study [18] in that a higher proportion of degenerate than non-degenerate discs expressed p16^INK4a^. Of the five non-degenerate samples (from PM source, mean age ± SD 45.8 ± 18.4 years), only two samples expressed p16^INK4a ^at copy numbers of 1.4 and 55.8 copies per 100 ng cDNA from individuals of 30 and 75 years of age, respectively. Eleven of the 12 degenerate samples (from both PM and surgical sources, 35.4 ± 12.7 years) expressed p16^INK4a ^with median and maximum copy numbers of 32.5 and 17,075 copies per 100 ng cDNA, respectively. qRT-PCR analysis demonstrated a significant correlation between caveolin-1 and p16^INK4a ^gene expression in the degenerate NP samples (*P *= 0.02) (Figure 5).

## Discussion

This study has demonstrated for the first time that cells from the NP of human IVDs express caveolin-1 and furthermore that caveolin-1 gene expression and protein expression are elevated in degenerate IVDs, but that this rise in caveolin-1 expression does not correlate with increasing age. This is consistent with a role for caveolin-1 in degenerative rather than age-induced changes in the NP.

Changes associated with tissue ageing and degeneration have been postulated to involve cellular senescence [41-43]. Two major categories of senescence are generally described in the literature as replicative senescence (RS) and SIPS. RS was first described by Hayflick in 1965 [44] and is widely regarded as one of the main mechanisms underlying the normal ageing process via reduction of telomere length to critical levels following cumulative population doublings. In addition, there are a number of reports describing premature induction of senescence as a result of cellular exposure to stress. Factors linked to the induction of SIPS vary widely, from DNA damage – for example, radiation (bovine aortic endothelial cells [45]), UV light (human fibroblasts [46] and human melanocytes [47]), chemical treatment (nasopharyngeal carcinoma cells [48] and human fibroblasts [49,50]), and oxidative stress (human fibroblasts [20,22,24] and human articular chondrocytes [19]) – to oncogenic protein overexpression (for example, ras in human fibroblasts [51]) and exposure to inflammatory cytokines such as IL-1 and tumour necrosis factor-α (human chondrocytes and fibroblasts [19,21,23]). Previous data from our laboratory described accelerated senescence (characterised by a variety of biomarkers, including reduced cell replication potential, elevated levels of the cell cycle inhibitor p16^INK4a^, increased SA-β-gal activity, and telomere erosion) in degenerate human IVDs compared with age-matched non-degenerate discs [18], suggesting that SIPS may be involved in IVD degeneration.

Caveolin-1 forms homodimers, or heterodimers with its family member caveolin-2, that insert into the plasma membrane of terminally differentiated cells [25]. The caveolin-1-rich areas termed caveolae and the caveolin proteins themselves are proposed to regulate cellular processes, including membrane traffic, signal transduction, and cellular senescence [25-28,52]. Caveolin-1 was investigated here due to its possible role in cellular senescence, in particular SIPS [19,31,52]. Here, we show that caveolin-1 gene expression and protein expression are increased during IVD degeneration, but not in a manner that is associated with increasing chronological age.

Moreover, we demonstrate a correlation between caveolin-1 and p16^INK4a ^gene expression. p16^INK4a ^is a cyclin-dependent kinase inhibitor that prevents retinoblastoma phosphorylation and arrests the cell cycle in the G_0_/G_1 _phase prior to entry into the synthesis phase [53,54]. Many studies have shown increased levels of p16^INK4a ^alongside the occurrence and maintenance of permanent growth arrest and senescence, including a rodent model of ageing [55-57]. Previous studies by our group and others strongly suggest a role for p16^INK4a ^in cellular senescence within degenerate tissue when compared with age-matched controls [18,58]. Furthermore, elevated p16^INK4a ^expression has been described in the premature senescence of human fibroblasts and leukaemic cells exposed to oncogenic ras and DNA double-strand breaks [51,59,60], strengthening the reports that p16^INK4a ^is a biological marker for senescence. The present study demonstrated that the increased expression of caveolin-1 seen in the degenerate NP positively correlated with gene expression for p16^INK4a^, suggesting that caveolin-1 expression is linked to the senescent phenotype observed in these cells.

The literature describes evidence linking cell exposure to stressful stimuli to both caveolin-1 expression and cellular senescence. In mouse NIH 3T3 fibroblasts, administration of subcytotoxic levels of H_2_O_2 _to experimentally mimic oxidative stress induced cellular senescence and increased caveolin-1 expression. Treatment with H_2_O_2 _in the presence of caveolin-1 antisense oligonucleotides reduced expression of senescence biomarkers, whereas transgenic overexpression of caveolin-1 induced SIPS [31]. In human endothelial cells, isolated from atherosclerotic patients and induced to senesce, caveolin-1 expression was correlated with senescence biomarkers and with expression of 4-hydroxynonenal expression (a marker of lipid peroxidation and thus oxidative stress) independently of an effect on telomere length [31]. These studies strongly support a role for caveolin-1 in SIPS induced by oxidative stress and this is further strengthened by work conducted on osteoarthritic articular chondrocytes. Administration of H_2_O_2 _to these chondrocytes induced cellular senescence via expression of the caveolin-1 protein, a mechanism reversed by antisense oligonucleotide-mediated downregulation of the caveolin-1 gene [19]. The same study demonstrated an identical role for the inflammatory cytokine IL-1β.

Articular chondrocytes and the degenerative process observed during osteoarthritis share many characteristics with IVD cells and IVD degeneration [12,43]. Interestingly, IVD cells are subjected to both oxidative stress and catabolic cytokines, which have been implicated in the induction of SIPS [19-22,24]. Work published by our group suggests that IL-1β not only is increased in degenerate discs but is an important factor involved in catabolic events during IVD degeneration, including decreased matrix production and increased MMP and ADAMTS expression [37,38,61,62]. Moreover, advanced glycation endproducts (AGEs) such as carboxymethyl-lysine (CML) and the receptor for AGEs (RAGE) have been localised to the NP of degenerate IVD [34-36]. CML is a tissue marker for accumulated oxidative stress [35]; therefore, its presence and that of its receptor RAGE are highly significant for both mechanisms underlying IVD degeneration and the likelihood that they could cause SIPS in human NP cells. Furthermore, RAGE has been localised to caveolin-1-rich membranes in endothelial cells [63]. This gives evidence, together with studies involving IL-1, that there are factors in the degenerate disc that may induce caveolin-1 expression and thus lead to the senescent phenotype described in IVD cells [16-18].

Caveolin-1-rich regions of the plasma membrane have been associated with several receptors and signalling molecules, predominantly through isolation of caveolae and colocalisation studies. These studies have highlighted a subset of proteins that are relevant to IVD degeneration and to SIPS. First, RAGE, described above, is known to regulate several intracellular signalling pathways, including the nuclear factor-kappa-B pathway, which is essential for the expression of MMPs present in the degenerate IVD [34,64]. Second, there is evidence suggesting that caveolin-1, β1 integrin, and urokinase plasminogen activator receptor (uPAR) colocalise in human articular chodrocytes [65]. uPAR has an integral role in plasmin activation and thereby promotes catabolic events through initiation of a proteolytic cascade through which matrix-degrading enzymes described in IVD degeneration such as MMPs are activated [66]. Both could conceivably be pathways via which elevated caveolin-1 levels exert aspects of the senescent cellular phenotype observed in IVD degeneration.

## Conclusion

This study has shown that caveolin-1 expression in human NP cells is linked to IVD degeneration and is associated with the senescent phenotype as depicted by increased expression of p16^INK4a^. Caveolin-1 expression was not linked to increasing chronological age, suggesting a role in accelerated degeneration which could be due to SIPS, rather than RS. Further work will elucidate the role of caveolin-1 in these related areas.

## Abbreviations

ABI = Applied Biosystems (Warrington, UK); ADAMTS = a disintegrin and metalloprotease with thrombospondin motifs; AF = annulus fibrosus; AGE = advanced glycation endproduct; CML = carboxymethyl-lysine; Ct = cycle threshold; DMEM + F-12 = Dulbecco's modified Eagle's medium and Ham's F-12 nutrient medium; gDNA = genomic DNA; IHC = immunohistochemistry; IL = interleukin; IVD = intervertebral disc; LBP = low back pain; MMP = matrix metalloproteinase; NP = nucleus pulposus; PCR = polymerase chain reaction; PDAR = pre-developed assay reagent; PM = post mortem; qRT-PCR = quantitative real-time reverse transcription-polymerase chain reaction; RAGE = receptor for advanced glycation endproducts; RS = replicative senescence; SA-β-gal = senescence-associated β-galactosidase; SD = standard deviation; SEM = standard error of the mean; SIPS = stress-induced premature senescence; TBS = Tris-buffered saline; uPAR = urokinase plasminogen activator receptor.

## Competing interests

The authors declare that they have no competing interests.

## Authors' contributions

SKH participated in the design of the study, performed the majority of the laboratory work and analysis, and drafted the manuscript. CLM helped to secure funding, participated in the design of the study and the interpretation of data, and assisted in the preparation of the final manuscript. JAH conceived the study, secured funding, contributed to the design and coordination of the study, and participated in the interpretation of data and extensive preparation of the final manuscript. All authors read and approved the final manuscript.
